# Re-balance of memory T cell subsets in peripheral blood from patients with CML after TKI treatment

**DOI:** 10.18632/oncotarget.20965

**Published:** 2017-09-16

**Authors:** Danlin Yao, Ling Xu, Jiaxiong Tan, Yikai Zhang, Shuai Lu, Mingde Li, Sichun Lu, Lijian Yang, Shaohua Chen, Jie Chen, Jing Lai, Yuhong Lu, Xiuli Wu, Xianfeng Zha, Yangqiu Li

**Affiliations:** ^1^ Key Laboratory for Regenerative Medicine of Ministry of Education, Institute of Hematology, Jinan University, School of Medicine, Jinan University, Guangzhou, China; ^2^ Department of Hematology, First Affiliated Hospital, Jinan University, Guangzhou, China; ^3^ Department of Clinical Laboratory, First Affiliated Hospital, Jinan University, Guangzhou, China

**Keywords:** memory T cells, TSCM, CML, TKI, Immunology and Microbiology Section, Immune response, Immunity

## Abstract

T cell immune surveillance is considered an important host protection process for inhibiting carcinogenesis. The full capacity of T cell immune surveillance is dependent on T cell homeostasis, particularly for central memory T (T_CM_) cells and stem cell memory T (T_SCM_) cells. In this study, distribution of T cell subsets in peripheral blood from 12 patients with chronic myeloid leukemia (CML) and 12 cases with CML in complete remission (CR) was analyzed using a multicolor flow cytometer, and 16 samples from healthy individuals (HIs) served as control. The proportion of CD8^+^ T_SCM_ and CD4^+^ and CD8^+^ T_CM_ cells were lower, while CD4^+^ effector memory T (T_EM_) cells and CD4^+^ and CD8^+^ terminal effector T (T_EF_) cells were higher in CML patients compared with HIs. Moreover, the proportion of CD8^+^CD28^-^ T cells, which were found to have the immune suppressive function, increased in the naive T (T_N_) cell and T_CM_ subsets in CML patients compared with HIs. Our study reveals that elimination of leukemia cells by treating with tyrosine kinase inhibitors (TKIs) restores the memory T cell distribution from a skewed pattern in CML patients who are under leukemia burden, indicating that leukemia-specific immune responses mediated by T cells might be induced and maintained in CML patients, however, these responsive T cells might gradually become exhausted due to the continued existence of leukemia cells and their environment; therefore, T cell activation using a different approach remains a key point for enhancing global T cell immunity in CML patients, even for those with CR status.

## INTRODUCTION

Anti-leukemia immunity plays an important role in protecting against the development of leukemia cells. Graft-*versus*-leukemia effects have been proven to have the power to eliminate minimal residual disease (MRD) in the setting of allogeneic stem cell transplantation (allo-SCT) and donor lymphocytes infusion (DLI) [1-3]. Other immunotherapeutic strategies under investigation include vaccination and adoptive T cell transfer, which have had some exciting results [3-5], but initial trial data have been disappointing [6]. Leukemia cells and the leukemia cell-induced immune suppressive microenvironment are likely to be responsible for failure in these immunotherapeutic approaches. For example, downregulation or overexpression of costimulation or activation molecules can interfere with the function and survival of immune cells, besides, exceptional secreted cytokines and exosomes from leukemia environment can disturb the metabolism and/or differentiation of immune cells [7-10]. In addition, several immune suppressive cells have been found to be increased in leukemia patients, such as regulatory T cells (Treg) [11] and myeloid-derived suppressor cells (MDSCs) [12, 13], and there is an imbalance in the Th1/Th2 ratio. Therefore, a better understanding of the impact of leukemia cells on the host immune system is critical for developing successful immunotherapy strategies.

CML is a clonal hematopoietic stem cell disorder characterized by the translocation t(9;22)(q34;q11.2), which results in the creation of the novel *BCR-ABL* fusion gene with abnormal tyrosine kinase activity [14-17]. Tyrosine kinase inhibitors (TKIs) such as imatinib mesylate (IM) is a targeted molecular drug that serves as frontline therapy for all phases of CML that works by binding to the tyrosine kinase domain of BCR-ABL and inhibiting its function [18]. TKIs are often well tolerated; however, TKI-based therapy is considered to be lifelong because rapid disease relapse often occurs upon drug discontinuation. As tyrosine kinases are key regulators of immune responses, long-term off-target effects on normal cells and tissues may raise concerns that immunosuppressive effects may occur by continued treatment with TKIs. Studies from other groups have shown that IM can reversibly inhibit T cell proliferation *in vitro*; however, the distribution of lymphocyte subsets and the proliferation and activation status of lymphocytes from imatinib-treated patients were similar to healthy controls in a study by Rohon et al. with the exception of a lower number of γδ T cells and a larger amount of CD45RO positive CD4^+^ T cells in PB [19]. It is well known that an adequate number of naïve and memory cells must exist to achieve lifelong protection against pathogens, particularly for T memory cells, which have the ability to rapidly transition from a quiescent state to a highly proliferative cytolytic population of effector cells upon antigen reexposure [20, 21]. Recently, a new subset of human memory T cells has been identified based on expression of the surface markers CD95 and CD122. These memory T cells possess a stem cell-like capacity to self-renew and differentiate into all subsets of memory and effector cells; thus, these cells were termed stem cell memory T cells (T_SCM_), and they have been revealed to be quite important for T cell immune reconstitution after HSCT and long-term immune surveillance [22].

Based on the importance of T cell memory and homeostasis in human immunity, in this study, we assayed the distribution of all of the memory T cell subsets in patients with CML and those who achieved CR by treatment with TKIs to assess the impact of this disease and TKIs on the patient T cell subsets as well.

## RESULTS AND DISCUSSION

**Figure 1:**
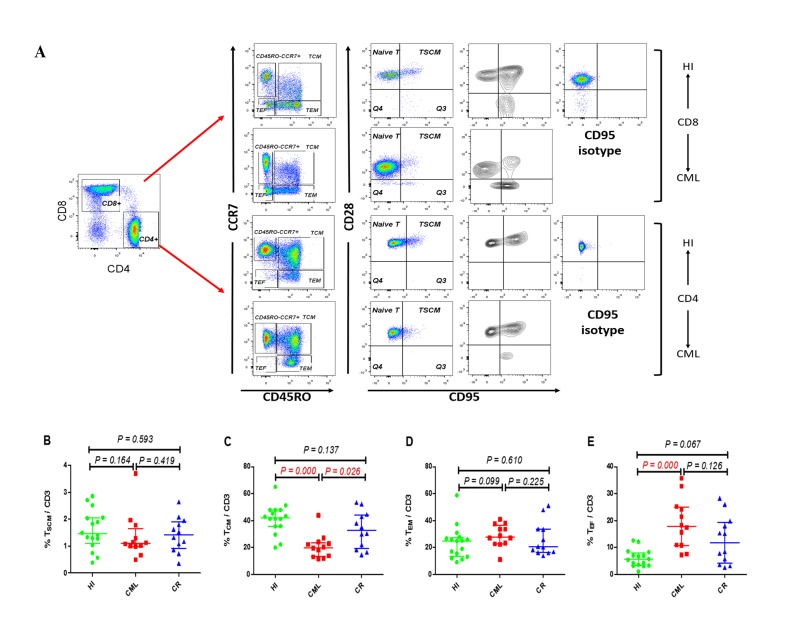
Gating strategy for identifying the CD4 and CD8 T cell subsets and the frequency of T_SCM_, T_CM_, T_EM_ and T_EF_ cells within CD3^+^ population (**A**) Gating strategy for the identification of CD4 and CD8 T cell subsets in one HI and one CML patient. CD45RO and CCR7 were used to divide the T cells into four subsets, and T_SCM_ cells were then further gated from the CD45RO^-^ CCR7^+^ population by CD95 and CD28 expression, and separation of the T_SCM_ cells from CD95^−^ T_N_ cells was performed with the help of gating CD95 on the CD4 or CD8 population and the CD95 FMO control; (**B** - **E**). Frequency of T_SCM_, T_CM_, T_EM_, and T_EF_ among CD3^+^ T cells from16 HIs, 9 patients with CML, and 9 cases with CML-CR. HI: healthy individuals, CML: chronic myeloid leukemia, CML-CR: CML achieved complete remission.

To compare the proportion of peripheral CD3^+^, CD4^+^, and CD8^+^ T cells in CML patients with those from HIs, as well as samples from CML-CR patients treated with TKIs, we used eight types of antibodies for analysis. The gating strategy is shown in Figure 1A. CD45^high^ lymphocytes expressing CD3 and CD4, or CD3 and CD8 were gated as CD3^+^, CD4^+^, and CD8^+^ T cells, respectively, and then divided into four populations by the expression of CD45RO and CCR7. Naïve T cells (T_N_) and T_SCM_ cells were defined as CD45RO^-^CCR7^+^, T_CM_ cells were CD45RO^+^CCR7^+^, T_EM_ cells were CD45RO^+^CCR7^-^, and T_EF_ cells were CD45RO^-^CCR7^-^. The T_SCM_ cells were further gated from the CD45RO^-^CCR7^+^ population by CD95 and CD28 expression, and with the exception of the T_SCM_ subset, the other subsets could be further analyzed for their expression of CD28 [23, 24].

**Figure 2:**
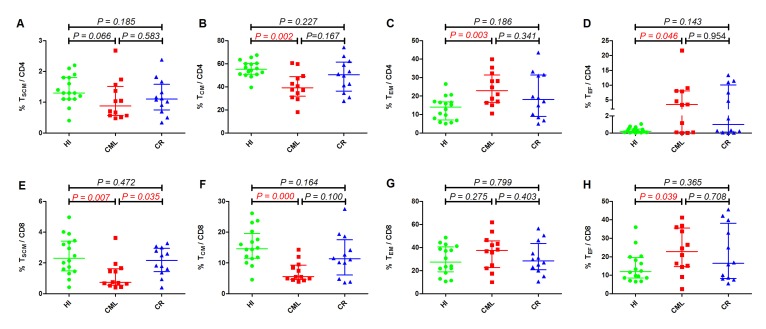
Frequency of TSCM, TCM, TEM and TEF among the CD4^+^ ( Top ) and CD8^+^ (Below) (**A**). T_SCM_/CD4^+^, (**B**). T_CM_/CD4^+^, (**C**). T_EM_/CD4^+^, (**D**). T_EF_/CD4^+^, (**E**). T_SCM_/CD8^+^, (**F**). T_CM_/CD8^+^, (**G**). T_EM_/CD8^+^, (**H**). T_EF_/CD8^+^. HI (n=16), CML (n=12), CML-CR (n=12). HI: healthy individuals, CML: chronic myeloid leukemia, CML-CR: CML achieved complete remission.

We first compared the CD3^+^ T_SCM_, T_CM_, T_EM,_ and T_EF_ subsets distribution in the three groups, and the results demonstrated that there was no difference between the groups for T_SCM_ and T_EM_ cells, while there was a distinct decreased proportion in the T_CM_ subset in the CML group (20.0%, *p* = 0.000); however, there was no significant difference in the CML-CR group (32.8%, *p* = 0.163) compared with HIs (42.1%) (Figure 1C). Accordingly, there was a significantly increased proportion of T_EF_ cells in the CML group (17.9%, *p* = 0.000) compared with HIs (5.6%) but not in the CML-CR group, which was examined at the same time (Figure 1E). Due to the different functions of the CD4^+^ and CD8^+^ T cells in immunity, we further analyzed the distribution of the different subsets in the CD4^+^ and CD8^+^T cell populations. Similarly, there was no difference in the proportion of CD4^+^ and CD8^+^ T_N_ cells between the CML or CML-CR groups and HIs. With the development of multicolor flow cytometry and high throughput sequencing, human memory T cells could be further classified into four subsets, and T_SCM_ and T_CM_ cells have the most potential for adoptive therapy because they can rapidly differentiate into the T_EM_ and T_EF_ subsets after antigen restimulation to control the invasion and spread of pathogens [25]. In this study, we found a dramatically decreased CD8^+^ T_SCM_ subset in the CML group (0.7%, *p* = 0.007) compared with HIs (2.3%). While this change was recovered in CML-CR patients (2.2%, *p* = 0.472), the CD4^+^ T_SCM_ population also demonstrated the same pattern, but the difference was not statistically significant (Figure 2A and 2E). For the T_CM_ proportion, both CD4^+^ and CD8^+^ T_CM_ cells were sharply decreased in the CML group (CD4^+^: 39.2%, *p* = 0.002; CD8^+^: 5.5%, *p* = 0.000), but they were nearly normalized in CR patients (CD4^+^: 50.4%, *p* = 0.227; CD8+:11.3% *p* = 0.164), when compared with HIs (CD4^+^: 55.1%; CD8^+^: 14.7%) (Figure 2B and 2F).

T_EM_ and T_EF_ cells are more differentiated subsets that can continually secrete cytokines (CD4) or cytotoxic molecules (CD8) to protect against infections and tumor cells when exposed to antigens [20, 25]. Here, we reported that the CD4 T_EM_ and T_EF_ cell subsets increased in the CML group (T_EM_: 22.9%, *p* = 0.003; T_EF_: 3.5%, *p* = 0.046) compared with HIs (T_EM_: 14.1%, T_EF_: 0.2%), but there was no significant difference between the CML-CR group and HIs (Figure 2C and 2D). Similar to the CD4 subset, the proportion of the CD8 T_EM_ and T_EF_ cell population also increased in the CML group (Figure 2G and 2H); however, the changes were only significant for the CD8 T_EF_ subset (CML: 22.8%, HI: 12.0%, *p* = 0.039). The T_EM_ and T_EF_ subsets were nearly normal in CR patients; however, some of the CR patients still had a higher level of the CD4 and/or CD8 T_EM_ and T_EF_ subsets, which may be associated with the degree of remission in these patients. It appears that the inclination toward more differentiated T cell subsets is not a unique characteristic of CML patients because circulating CD8^+^ T cells skewing from naïve to T_EF_ and T_EM_ subsets accompanying the shrunken pool of T_CM_ was also reported in head and neck squamous cell carcinoma (HNSCC) patients; moreover, activated T_EM_ and T_EF_ cells were dramatically increased in patients with advanced-stage disease [20, 21]. These results indicated that T cells in CML patients have the capacity to more quickly differentiate into effector; however, it remains unclear whether these cells maintain full immune function. Moreover, TKI treatment can recover T cell subsets.

**Figure 3:**
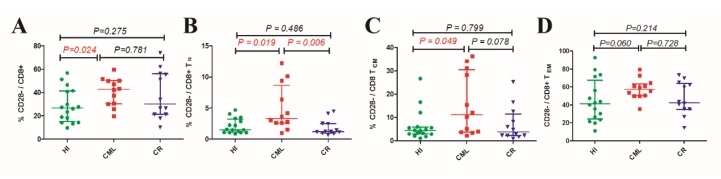
Increased CD28^-^ T cell in CD8 population and their increased expression in CD8 T cell subsets (**A**). Frequency of CD28^-^ cells in total CD8^+^ population. (**B**). CD28^-^ cell frequency in CD8^+^T_N_ subsets. (**C**). CD28^-^ cell frequency in CD8^+^ T_CM_ subsets. (**D**). CD28^-^ cell frequency in CD8^+^ T_EM_ subsets. HI (n=16), CML (n=12), CML-CR (n=12). HI: healthy individuals, CML: chronic myeloid leukemia, CML-CR: CML achieved complete remission.

It has been reported that most of the increased T_EF_ and T_EM_ cells have a CD28 negative phenotype, and these CD28^−^ T_EF_ cells demonstrate a lack of TCRζ chain expression or overexpression of early apoptosis markers. After tumor excision or treatment with chemotherapy, the skewed T cell subsets and CD28 expression have been reported to recover to normal [21]. We also found that there was an increased percentage of CD28^-^ population in total CD8 (CML: 46.3%, HI: 26.7%, *p* = 0.024) (Figure 3 A) but not in CD4 (CML: 7.1%, HI: 3.7%, *p* = 0.186) T cell subset of CML patients, further analysis revealed that those increased percentage of CD28 negative population mainly distributed in the T_N_, T_CM_ subsets (Figure 3B to 3D), meanwhile, CD28^-^ population distribution in CR group showed almost the same with HI group, not matter in the total CD8 subset or the CD8^+^ memory T cell subsets (Figure 3A to 3D ). Recently, CD8^+^CD28^−^ cells have been defined as CD8^+^ Treg cells and have been observed to have an immune regulatory function in patients who have undergone successful organ transplantation or alloanergized HLA mismatched bone marrow grafts [26] or who were suffering from autoimmune diseases and cancers [27-29]. These cells can mediate immune suppression by directly contacting antigen presenting cells (APCs) and upregulating inhibitory receptors (ILT3 and ILT4) on APCs or by secreting IL-10 [30]. Importantly, CD8^+^CD28^−^ T cells isolated from healthy donors do not show immunosuppressive properties [30]. Hence, it would appear that CD8^+^CD28^−^ Treg cells are induced in the periphery following disturbances in immune homeostasis [31], and *in vitro* experiments have revealed that the CD28^−^ T cell population can be induced by the continued stimulation of CD28^+^ T cells with tumor cells, which may be related to improper co-stimulation signals provided by tumor cells. Similarly, Rifca Le Dieu reported an increase in the absolute number of CD3^+^CD56^+^ cells ( though not natural killer T (NK-T) cells) in the blood of patients with acute myeloid leukemia (AML) compared with healthy controls, and these cells more frequently expressed CD57 and less frequently expressed CD28 [22].

In this study, we report for the first time the characteristics of the skewed T cell subsets in the PB of CML patients with leukemia burden, which is similar to that found in HNSCC and AML patients, and the abnormal distribution pattern nearly recovered after CR by IM treatment. This discovery is also similar with the finding that excising a tumor could restore the TLI subset distribution in HNSCC. These results suggest that direct contact and stimulation with tumor cells will result in a T cell response and differentiation into the T_EF_ and T_EM_ populations; however, the continued imbalanced in T cell distribution yields protection potential for naïve T cells and a long-term protective capacity for the T_SCM_ and T_CM_ subsets. Moreover, chronic tumor-associated antigen stimulation may result in a dysfunction in the T_EF_ and T_EM_ population similar to that found with chronic virus infection. Although we do not have direct evidence of T cell dysfunction in present study, this evidence may be indirectly provided in our previous studies and other reports that have shown low TCR ζ chain expression and high expression of program death 1 (PD-1) on the T cells of CML patients [23-28]. Further investigation will focus on determining the ways in which dysfunction occurs in the different T cell subsets and revealing the mechanisms underlying it. In addition, we also assayed for the first time the T_SCM_ distribution in the PB of CML patients in this study, and our results indicate that CD8^+^ T_SCM_ combined with T_CM_ loss may greatly contribute to patient infection susceptibility, especially for fungus and virus infection, the speculation is based on T_SCM_ and T_CM_ playing an important role in the adaptive immune response upon reinfection, as well as that CML patients showed more susceptibility to Cryptococcus and other infection diseases compare with healthy individuals [22, 25, 32, 33]

**Figure 4:**
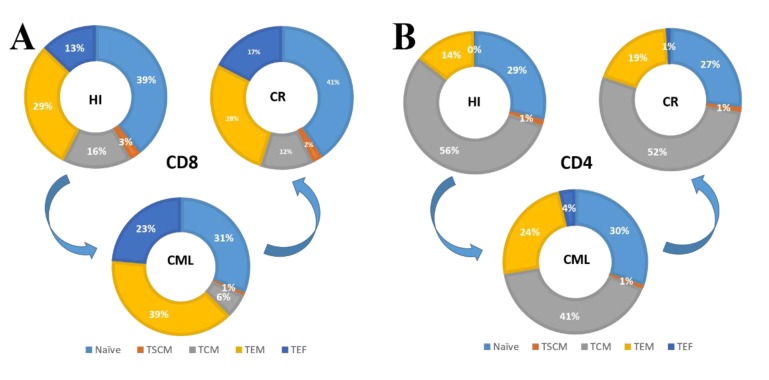
Pie charts summarize the distribution changes in the CD4 and CD8 T cell subsets **A.** The CD8^+^ T cell subsets changes from HIs to CML patients and then recovers in CML-CR patients; **B.** CD4^+^ T cell subsets change from HIs to CML patients and then recovers in CML-CR patients.

In summary, we characterized for the first time a skewed distribution of the CD3 T cell subsets (decreased T_CM_ combined with increased T_EF_) in CML patients, but this became normalized in patients with CML-CR after TKI therapy. Further analysis of the CD4 and CD8 sub-populations revealed that the reduced T cell subsets were CD8^+^ T_SCM_ cells and CD4 and CD8 T_CM_ cells. In contrast, the accumulated T cell subsets were CD4 T_EM_ and CD4 and CD8 T _EF_ subsets. The imbalance in memory CD4^+^ and CD8^+^ T cell subsets is schematically shown in Figure 4. Based on the discovered T cell dysfunction in CML patients reported by previous studies, we hypothesize that most of the accumulated T_EM_ and T_EF_ cells were exhausted to some degree, while the different degree of accumulation and exhaustion may have a direct impact on the patient response to TKIs and prognoses, which is worth further investigation. Overall, the findings suggested that these responsive T cells might gradually become exhausted due to the continued existence of leukemia cells and their environment; therefore, T cell activation using a different approach remains a key point for enhancing global T cell immunity in CML patients, even for those with CR status.

## MATERIALS AND METHODS

### Samples

PB samples were obtained with consent from 12 CML patients, including 7 cases in chronic phase (CP), 2 cases in acceleration phase (AP) and 3 cases in blast crisis (BC), (7 males and 5 females with a median age of 37 years); 12 CML patients who achieved complete remission (4 males and 8 females with a median age of 41 years) by treating with Imatinib (200 to 400 mg/day), the length of treatment was different for each patient, from 1 month to 5 years, there were 3 CR patients also assayed at the time of diagnosis which included in the CML group, the response degree in each patient was different, which from complete hematologic response (CHR) to Major molecular response (MMR); 16 healthy volunteers (7 males and 9 females with a median age of 36.5 years). The characteristics of the patients and healthy volunteers are summarized in Table 1. All of the procedures were conducted according to the guidelines of the Medical Ethics Committees of the Health Bureau of the Guangdong Province in China, and ethical approval was obtained from the Ethics Committee of the Medical School of Jinan University.

**Table 1:** Characteristics of healthy volunteers, patients with CML, and CML patients in CR treated with TKIs

Variable*	HI group	CML group		CR group
Age (Year)	36.5 (21∼52; 16)	37 (19∼61; 12)		41 (19∼61; 12)
Male/Female	7/9	7/5		4/8
WBC (×10^9^/L)	/	170 (33∼523; 12)		6 (3∼9; 12)
PLT (×10^9^/L)	/	389 (52∼853; 12)		179 (84∼573; 12)
Blast and Promyelocytes in blood (%)	/	CP	1 (0∼7; 7)	/
		AP+BC	10 (1∼67; 5)	

WBC = white blood cell, PLT= platelet, CP = chronic phase, AP = acceleration phase, BC = blast crisis. *Continuous variables are expressed as median (range; number of cases).

### Immunophenotyping analysis by flow cytometry

The strategy for T_SCM_ analysis followed the protocol of Lugli E [23]. Cell surface staining for flow cytometry was performed with the following: CD45-APC (C1.7), CD3-FITC (HIT3a), CD4-APC-H7 (RPA-T4),CD8-Percp-Cy5.5 (SK1), CCR7-BV421 (150503), CD45RO-BV510 (UCHL1), CD28-PE (CD-28.2), CD95-PE-Cy7 (DX2), BV510 Isotype Control (G155-178) and PE-Cy7 Isotype Control (MOPC-21). Extracellular staining was performed according to the manufacturer’s instructions. Five microliters of CCR7-BV421 fluorescent antibody was incubated with approximately 200µl peripheral blood without plasma at 37°C for 15 minutes in the dark. Redundant fluorescent antibodies were eliminated by washing with 2 ml Cell Staining Buffer followed by centrifugation at 350g for 5 minutes, and the supernatant was discarded. Cells were then resuspended in 50µl antibody cocktail and incubated at room temperature for an additional 15 minutes in the dark. Then, 2ml 1X Red Blood Cell (RBC) Lysis Buffer (BD, Biosciences, USA) was added to suspend the stained cells, which were then incubated in dark at room temperature for 20 minutes. After centrifugation for 5 minutes at 350 g, the supernatant was discarded. Finally, the cells were washed twice with 2 ml Cell Staining Buffer by centrifugation at 350 g for 5 minutes, the supernatant was discarded and the samples were suspended in 0.5 ml staining buffer for analysis by flow cytometry. A total of 80,000 to 100,000 CD3^+^ cells were analyzed with a BD Verse flow cytometer (BD, Biosciences, USA), and data analysis was performed using FlowJo software.

### Statistical analyses

All data are represented as medians, and statistically significant differences between the different T cell populations as well as between CD8^+^CD28^-^ T cells were analyzed by the Mann-Whitney U test for nonparametric values. Calculations were performed using GraphPad Prism version 4.00 software.

## Abbreviations

CML: Chronic myeloid leukemia
CR: Complete remission
HSCT: Hematopoietic stem cell transplantation
PB: Peripheral blood
PBMCs: Peripheral blood mononuclear cells
MRD: minimal residual disease
T_N_: naive T cells
T_CM_: central memory T cell
T_SCM_: stem cell memory T
T_EM_: effector memory T cells
T_EF_: terminal effector T
DLI: donor lymphocytes infusion
Treg: regulatory T cells
MDSCs: myeloid-derived suppressor cells
TKIs: Tyrosine kinase inhibitors
IM: imatinib mesylate
HNSCC: head and neck squamous cell carcinoma
APCs: antigen presenting cells
NKT: natural killer T
AML: acute myeloid leukemia
PD-1: program death 1
WBC: white blood cell
PLT: platelet
CP: chronic phase
AP: acceleration phase
BC: blast crisis
